# Iatrogenic injury of posterolateral structures during femoral tunneling in anterior cruciate ligament reconstruction: A cadaveric study

**DOI:** 10.1016/j.amsu.2020.09.012

**Published:** 2020-09-09

**Authors:** Sholahuddin Rhatomy, Jaka Fatria Yudhistira, Noha Roshadiansyah Soekarno, Riky Setyawan

**Affiliations:** a Department of Orthopaedics and Traumatology, Dr. Soeradji Tirtonegoro General Hospital, Klaten, Indonesia/ Faculty of Medicine, Public Health, and Nursing, Universitas Gadjah Mada, Yogyakarta, Indonesia; b Department of Orthopaedics and Traumatology, Radjak Hospital, Salemba, Jakarta, Indonesia; c Department of Orthopaedics and Traumatology, Dr. Chasbullah Abdulmadjid General Hospital, Bekasi, Indonesia; d Soeradji Tirtonegoro Sport Center and Research Unit, Dr. Soeradji Tirtonegoro General Hospital, Klaten, Indonesia/ Faculty of Medicine, Public Health, and Nursing, Universitas Gadjah Mada, Yogyakarta, Indonesia

**Keywords:** Anterior cruciate ligament reconstruction, Femoral tunneling, Iatrogenic injury, Posterolateral knee structure, Trans portal technique

## Abstract

**Objective:**

Creating Femoral tunnel in Anterior Cruciate Ligament (ACL) reconstruction can be done through some portal. Transportal technique commonly preferred by Orthopedic Surgeon. However, this technique may possess some iatrogenic injury to knee structure especially in the posterolateral during the drilling. This study aim is to describe the most susceptible injured posterolateral structure of the knee during femoral tunneling from trans portal technique Anterior Cruciate Ligament (ACL) reconstruction.

**Methods:**

Twenty knees from ten cadavers was examined. Anterior Cruciate Ligament (ACL) reconstruction was simulated using the trans portal technique. Femoral drilling was performed with knee in 120 flexion. The cadaver was dissected to identify the injured posterolateral structure during femoral tunneling. The data collected was anatomy structure and the wound size.

**Results:**

All Twenty knees were included in the study. 90% of injured structure was iliotibial band. The next most common was plantaris (50%) dan gastrocnemius (45%) muscle. The other was biceps femoris muscle (20%). The least common injury was vastus lateralis (5%). No injury was observed in Lateral Collateral Ligament (LCL), popliteus tendon, and peroneal nerve.

**Conclusion:**

Transportal technique during femoral drilling in Anterior Cruciate Ligament (ACL) reconstruction does do damage to some of the posterolateral structure. The injury was considered minimal and may not result in function deterioration. There is also no risk to common peroneal nerve.

## Highlights

• Iatrogenic Injury.
• Posterolateral Structures.
• Femoral Tunneling.
• Anterior Cruciate Ligament Reconstruction.
• Cadaveric Study.

## 1 Introduction

Anterior Cruciate Ligament (ACL) rupture is one of the most common knee sports injury. Research has been continuously done on the ACL reconstruction to achieve better outcome in terms of closer to the anatomical properties and minimal iatrogenic injury. The ultimate goal is the knee stability and the ability to return to sports [1,2].

There has been various technique to performed ACL reconstruction. Two familiar procedures are widely used in femoral tunnel drilling. They are transtibial technique and anteromedial portal (trans portal) technique [3]. Anatomic femoral tunnel position is difficult to achieved using transtibial technique. According to study by Alentorn-Geli et al. was shown that transtibial technique required longer time to recovery because it gave higher anteroposterior and rotational knee stability [4]. Some orthopedics choose another technique to perform femoral drilling [3,5].

Trans portal technique, recently most popular procedure because can create anatomic femoral tunnel. However, it might injured the posterolateral knee structure and the common peroneal nerve [6]. It is estimated that 10–20% of patients after ACL reconstruction still have persistent pain and instability [4,6]. This study goal is to identify and measure the anatomical structures that injured in the posterolateral knee during the procedure of femoral tunnel drilling. We hypothesize that there is an increased risk of direct injury to the common peroneal nerve and posterolateral structure.

## 2 Methods

This was descriptive study on the cadaver. Ten cadavers were obtained. Twenty knees (right and the left) was studied. The inclusion criteria were knee with normal condition. The exclusion criteria are obvious knee deformity, injury of the knee, and surgery scar around the knee region, fracture. All of the cadavers were fulfilled the inclusion criteria and none were excluded.

**Fig. 1:**
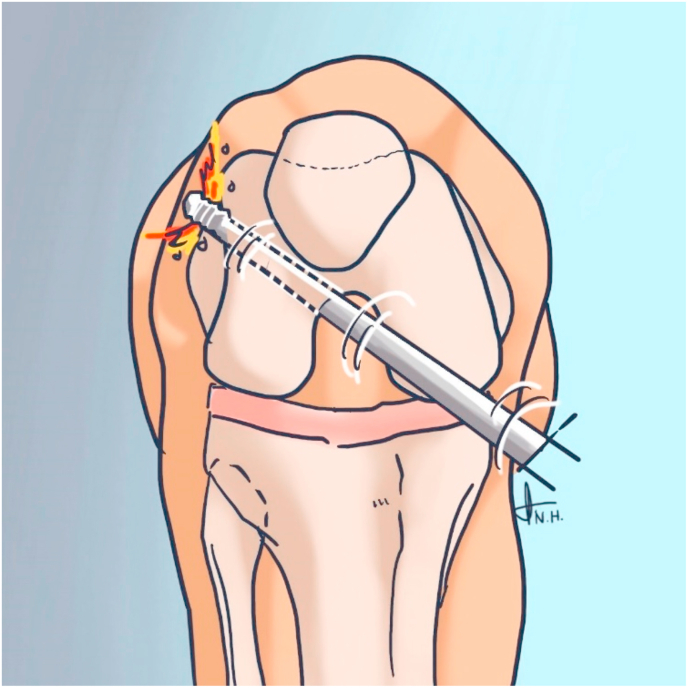
Additional drilling through the far cortex.

The ACL reconstruction was done by single Orthopedic Surgeon. Each knee had a 5 mm surgical incision for each anterolateral, anteromedial and accessory anteromedial portal. In 120° knee flexion (using goniometer), femoral tunneling was performed with 2.7 mm guide passing pin and 4.5 mm button reamer (Smith and Nephew instrument). The direction was aimed at 10 o'clock position in right knee and 2 o'clock in left knee around 2 mm from posterior femoral lateral condyle (using endoscopic femoral aimer arm offset, smith and nephew). Passing pin was drilled through skin. Additional drilling with button reamer was performed through the far cortex of the femur, this procedure according zelle et al. [7] (Fig. 1).

**Fig. 2:**
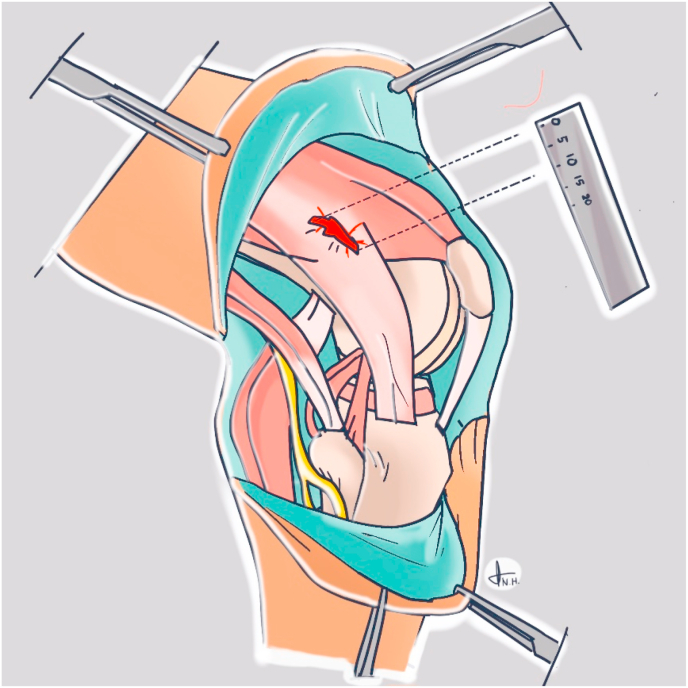
Identification and measurement of posterolateral knee iatrogenic injury during femoral tunneling.

To observe the injured structure, incision was made about 15 cm on the skin around exit point of the passing pin. Outside-in identification was done thoroughly from the skin to the cortex of the posterolateral knee breached by button reamer. Data collection was any posterolateral structure injured during the procedure and the size of injury. The size of injury was measured in millimeter (Fig. 2). Data analysis was performed using a SPSS® software version 25.0 by IBM Chicago, USA.

## 3 Results

There were 20 knees included in this study from 10 cadavers. The highest rate of injury was iliotibial band (90%) The next most common was plantaris (50%) dan gastrocnemius muscle (45%). The other was biceps femoris muscle (20%). The least common injury was vastus lateralis (5%). No injury was observed in Lateral Collateral Ligament (LCL), popliteus tendon, and peroneal nerve (Table 1).

**Table 1:** Rate of iatrogenic injury of posterolateral knee structures.

Anatomical Structures	Injury	
	Injury	%
Iliotibial Band	18	90
Plantaris Muscle	10	50
Lateral Gastrocnemius Muscle	9	45
Biceps Femoris Muscle	4	20
Vastus Lateralis Muscle	1	5
Popliteus muscle	0	0
Lateral Collateral Ligament	0	0
Peroneal nerve	0	0

The largest wound size was found in plantaris muscle with 5.0 mm diameter and the smallest wound size was found in iliotibial band with 2.0 mm (Table 2).

**Table 2:** Mean wound size of posterolateral knee structure iatrogenic injury.

Anatomical Structures	Mean Wound Size (mm)
Plantaris Muscle	5.0
Lateral Gastrocnemius Muscle	4.5
Vastus Lateralis Muscle	3.0
Biceps Femoris Muscle	2.3
Iliotibial band	2.0

## 4 Discussion

Our results confirmed that neurovascular structures were not injured when the femoral drilling was performed in standard fashion of 120° knee flexion. The most common injured structure was iliotibial band with 90% of incidence but it had the smallest wound size with 2.0 mm. Iliotibial band should be preserved to improve patient's perception of pain and give faster recovery [8]. Another study by Kawaguchi et al. stated that there was pain in posterolateral side of the knee after ACL reconstruction due to irritation of iliotibial band [9]. Cadaveric study by Hall et al.’s showed that there was no iatrogenic injury to peroneal nerve during guide wire drilling in knee flexion 70°, 90° and 120°. However, the risk of peroneal injury was increased in knee flexion less than 120°^4,6^, This supports the recommendations of Zelle et al. to keep the knee flexed to at least 110° to protect the peroneal nerve [7]. In my study the distance peroneal nerve from the drill hole is more than 8 mm.

In our study, we found that there were two structures that had mean wound size more than 3.0 mm. These structures were plantaris muscle with 5.0 mm and lateral gastrocnemius muscle with 4.5 mm. This wide wound size was probably caused by button reamer drilling that breached the far cortex of femur. Plantaris muscle was injured in 50% of the knees and lateral gastrocnemius muscle in 45% of the knees. The uninjured plantaris muscle or lateral gastrocnemius muscle were probably because the button reamer breached more proximal than the muscle origin. Injury to plantaris muscle and gastrocnemius muscle can produce pain in calf and plantarflexion and dorsiflexion movement [10].

In our study, we found that 20% injury was occurred in biceps femoris muscle at 120° knee flexion position. Hall et al. found that iatrogenic injury of biceps femoris was due to passing (guide) pin drilling in 40% of samples with 70° knee flexion, but no injury of biceps femoris in knee flexion more than 90°^6^.

This iatrogenic injury was only minor injury with less than 50% muscle tear that will be resolved after 3–4 weeks. Management of this type of injury usually well respond with conservative treatment. “PRICE” (Protection, Rest, Ice, Compression and Elevation) should be done for 2 days post-operative and should be continued with rehabilitation. These principles are also in synergy with post ACL Reconstruction rehabilitation protocols [11,12].

Further research is needed to determine the most optimum angle of knee flexion to minimize iatrogenic injury of the posterolateral knee structure when drilling the femoral tunnel while still maintaining the correct ACL footprint without sacrificing the safety of the peroneal nerve. Development of a safer instrumentation system for femoral tunneling using trans portal technique is also needed.

## 5 Conclusion

Transportal technique during femoral drilling in ACL reconstruction does do damage to some of the posterolateral structure. The injury was considered minimal and may not result in function deterioration. There is also no fatal risk to the neurovascular.

## Funding resource

This research has no funding resource.

## Declaration of competing interest

There is no conflict of interest to declare.
